# 4-Hy­droxy­methyl-10-meth­oxy-17,22-dioxapenta­cyclo­[21.2.2.2^13,16^.1^3,7^.0^11,30^]triaconta-1(25),3,5,7(30),8,10,13,15,23,26,28-undeca­ene-2,12-dione acetone monosolvate

**DOI:** 10.1107/S1600536812033521

**Published:** 2012-07-28

**Authors:** Daichi Hijikata, Kosuke Sasagawa, Sayaka Yoshiwaka, Akiko Okamoto, Noriyuki Yonezawa

**Affiliations:** a Department of Organic and Polymer Materials Chemistry, Tokyo University of Agriculture & Technology, Koganei, Tokyo 184-8588, Japan

## Abstract

In the title compound, C_30_H_26_O_6_·C_3_H_6_O, the *syn*-oriented benzoyl groups are nearly parallel to each other; the dihedral angle between their benzene rings is 15.9 (1)°. They form dihedral angles of 72.5 (1) and 84.3 (1)° with the naphthalene system. In the crystal, mol­ecules are linked into a three-dimensional architecture by C—H⋯O and C—H⋯π inter­actions.

## Related literature

For electrophilic aromatic aroylation of the naphthalene core, see: Okamoto & Yonezawa (2009 ▶); Okamoto *et al.* (2011 ▶). For applications of related mol­ecules, see; Okamoto *et al.* (2012 ▶). For the structures of closely related compounds, see: Hijikata *et al.* (2010 ▶); Mitsui *et al.* (2010 ▶); Sasagawa *et al.* (2011 ▶); Watanabe *et al.* (2010 ▶).
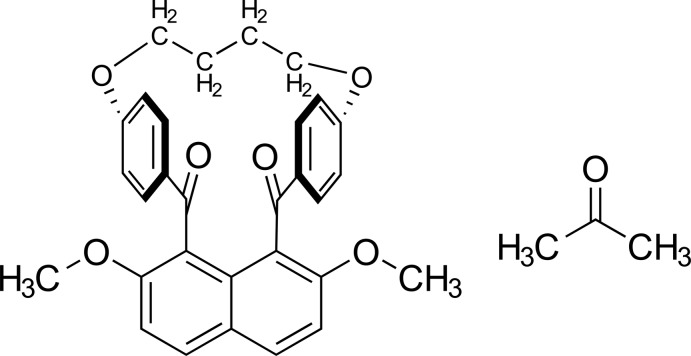


## Experimental

### 

#### Crystal data

C_30_H_26_O_6_·C_3_H_6_O
*M* *_r_* = 540.59
Orthorhombic, 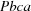

*a* = 15.4948 (3) Å
*b* = 16.1272 (3) Å
*c* = 22.4430 (4) Å
*V* = 5608.23 (18) Å^3^
*Z* = 8
Cu *K*α radiation
μ = 0.73 mm^−1^
*T* = 193 K
0.50 × 0.45 × 0.40 mm

#### Data collection

Rigaku R-AXIS RAPID diffractometer
Absorption correction: numerical (*NUMABS*; Higashi, 1999 ▶) *T*_min_ = 0.712, *T*_max_ = 0.759
99441 measured reflections
5132 independent reflections
4829 reflections with *I* > 2σ(*I*)
*R*_int_ = 0.021

#### Refinement

*R*[*F* ^2^ > 2σ(*F* ^2^)] = 0.037
*wR*(*F* ^2^) = 0.098
*S* = 1.05
5132 reflections
366 parameters
H-atom parameters constrained
Δρ_max_ = 0.20 e Å^−3^
Δρ_min_ = −0.19 e Å^−3^

### 

Data collection: *PROCESS-AUTO* (Rigaku, 1998 ▶); cell refinement: *PROCESS-AUTO*; data reduction: *CrystalStructure* (Rigaku, 2010 ▶); program(s) used to solve structure: *Il Milione* (Burla, *et al.*, 2007 ▶); program(s) used to refine structure: *SHELXL97* (Sheldrick, 2008 ▶); molecular graphics: *ORTEPIII* (Burnett & Johnson, 1996 ▶); software used to prepare material for publication: *SHELXL97*.

## Supplementary Material

Crystal structure: contains datablock(s) I, global. DOI: 10.1107/S1600536812033521/ld2069sup1.cif

Structure factors: contains datablock(s) I. DOI: 10.1107/S1600536812033521/ld2069Isup2.hkl

Supplementary material file. DOI: 10.1107/S1600536812033521/ld2069Isup3.cml

Additional supplementary materials:  crystallographic information; 3D view; checkCIF report

## Figures and Tables

**Table 1:** Hydrogen-bond geometry (Å, °) *Cg* is the centroid of the C12–C17 ring.

*D*—H⋯*A*	*D*—H	H⋯*A*	*D*⋯*A*	*D*—H⋯*A*
C3—H3⋯O2^i^	0.95	2.47	3.3241 (15)	150
C6—H6⋯O1^ii^	0.95	2.38	3.3245 (16)	172
C7—H7⋯O3^ii^	0.95	2.59	3.3910 (17)	143
C14—H14⋯O5^iii^	0.95	2.40	3.3328 (15)	169
C21—H21⋯O1*S* ^iv^	0.95	2.54	3.482 (2)	172
C2*S*—H2*S*2⋯*Cg* ^v^	0.98	2.86	3.830 (2)	171

Symmetry codes: (i) 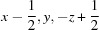; (ii) 
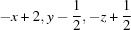
; (iii) 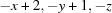; (iv) 
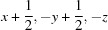
; (v) 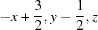.

**Fig. 1.**
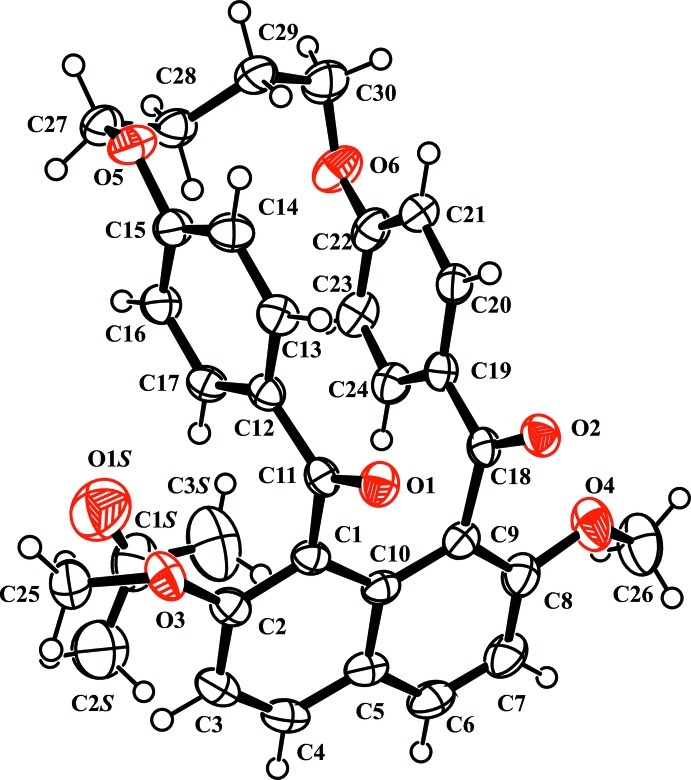
The molecular structure of the title compound with 50% probability displacement ellipsoids

**Fig. 2.**
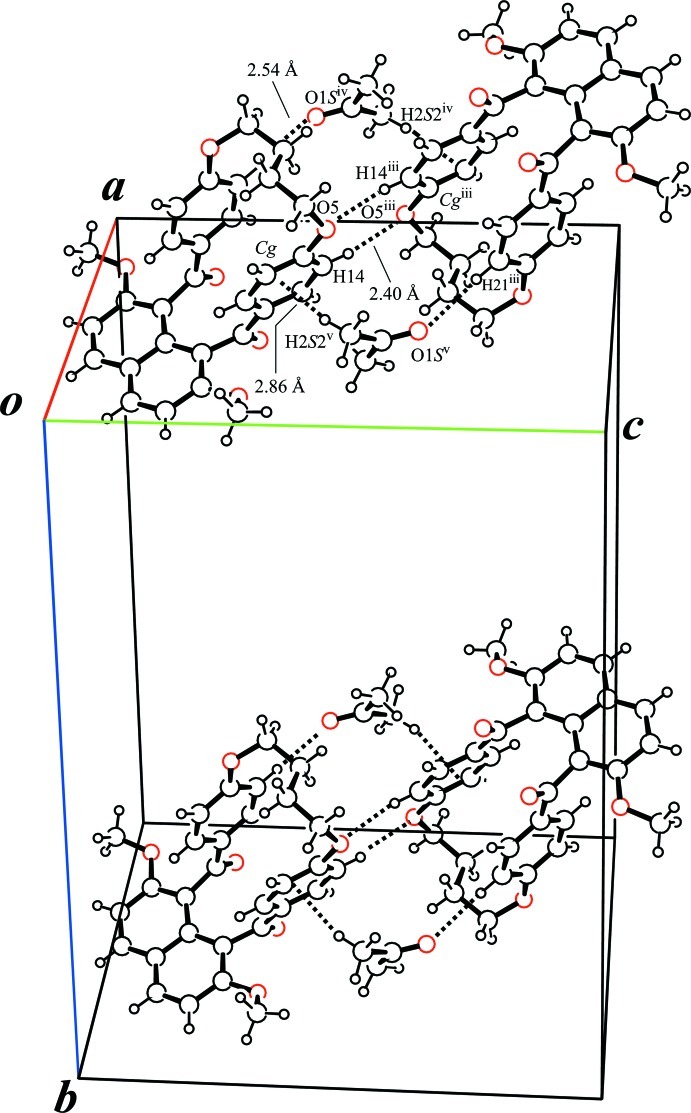
The dimeric associates of title compound. The C—H···O and C—H···π interactions are shown as dashed lines.

## supplementary crystallographic information

### Comment

In the course of our study on electrophilic aromatic aroylation of 2,7-dimethoxynaphthalene, *peri*-aroylnaphthalene compounds have proven to be formed regioselectively with the aid of suitable acidic mediators (Okamoto & Yonezawa, 2009; Okamoto, Mitsui *et al.*, 2011). As one of applications, the authors have integrated the resulting molecular unit to poly(ether ketone)backbone *via* nucleophilic aromatic substitution polycondensation (Okamoto *et al.*, 2012). Furthermore we have also reported the crystal structures of several 1,8-diaroylated naphthalene analogues exemplified by (2,7-dimethoxynaphthalene-1,8-diyl)bis(4-fluorobenzoyl)dimethanone (Watanabe *et al.*, 2010) and [8-(4-butoxybenzoyl)-2,7-dimethoxynaphthalen-1-yl](4-butoxyphenyl)methanone (Sasagawa *et al.*, 2011). These molecules have essentially same non-coplanarly features. The aroyl groups at the 1,8-positions of the naphthalene rings in these molecules are twisted in almost perpendicular fashion, but the benzene ring moieties of the aroyl groups tilt slightly toward the *exo* sides of the naphthalene rings. On the other hand, 1,8-bis(4-chlorobenzoyl)-7-methoxynaphthalene-2-ol ethanol monosolvate (Mitsui *et al.*, 2010) and 2,7-dimethoxy-1,8-bis(4-phenoxybenzoyl)naphthalene (Hijikata *et al.*, 2010) have apparently different spatial organizations. The aroyl groups attached to the naphthalene ring are oriented in the same directions. As a part of our continuous study on the molecular structures of this kind of molecules, the X-ray crystal structure of the title compound containing a 1,8-diaroylenenaphthalene moiety is discussed in this article.

The crystal packing is stabilized by intermolecular C—H···O hydrogen bonding between the oxygen atom (O2) of the carbonyl group of the adjacent molecule and one hydrogen atom (H3) on the naphthalene ring along the *a* axis (C3—H3···O2^i^= 2.47 Å; Table 1). Furthermore, two intermolecular C—H···O interactions, between the oxygen atom (O3) of the methoxy group and one hydrogen atom (H7) on the naphthalene ring, and between the oxygen atom (O1) of the carbonyl group and one hydrogen atom (H6) on the naphthalene ring, are observed along the *c* axis (C7—H7···O3^ii^= 2.59 Å, C6—H6···O1^ii^= 2.38 Å; Table 1). Moreover, the title compounds and acetones are linked by two C—H···O interactions and C—H···π interaction forming a three-dimensional architecture. The C—H···O interactions (C14—H14···O5^iii^= 2.40 Å, C21—H21···O1*S*^iV^= 2.54 Å; Fig. 2 and Table 1) and the C—H···π interaction (C2*S*^v^—H2*S*2^v^···*Cg*= 2.86 Å; Fig. 2 and Table 1) also contribute to the stabilization of the molecular conformation and crystal structure.

### Experimental

The title compound was prepared by S_N_2 reaction of 1,8-bis(4-hydroxybenzoyl)-2,7-dimethoxynaphthalene (1.0 mmol, 428 mg) with 1,4-dibromobutane (1.0 mmol, 215 mg) in *N*,*N*-dimethylacetamide (DMAc; 25.0 ml) with potassium carbonate (5.0 mmol, 691 mg). [The precursor, 1,8-bis(4-hydroxybenzoyl)-2,7-dimethoxynaphthalene, was obtained *via* S_N_Ar reaction of 1,8-bis(4-fluorobenzoyl)-2,7-dimethoxynaphthalene with sodium hydroxide.] After the reaction, the mixture was stirred at 333 K for 48 h, it was poured into water and extracted with CHCl_3_. The combined extracts were washed with 2*M* aqueous NaOH followed with brine. The organic layers were dried over anhydrous MgSO_4_. The solvent was removed under reduced pressure to give the crude product, which was purified by column chromatography (silica gel, CHCl_3_; isolated yield 47%). The pure product was crystallized from acetone to yield single crystals.

^1^H NMR δ (300 MHz, CDCl_3_): 1.78–1.93(4*H*, m),3.72(6*H*, s), 4.10–4.27(4*H*, m) 6.31(2*H*, dd, *J*=8.5, 2.4 Hz), 6.63(2*H*, dd, *J*=8.9, 2.4 Hz), 6.88(2*H*, dd, *J*=8.5, 2.0 Hz), 7.20(2*H*, d, *J*=8.9 Hz), 7.87(2*H*, dd, *J*=8.9, 2.0 Hz), 7.92(2*H*, d, *J*=8.9 Hz) p.p.m.

^13^C NMR δ (75 MHz, CDCl_3_): 22.49, 56.59, 66.80, 111.15, 113.77, 115.45, 121.81, 125.10, 128.81, 129.93, 131.24, 131.69, 133.82, 156.01, 160.90, 193.86 p.p.m.

IR (KBr): 1668 (C=O), 1600, 1509, 1460 (Ar, naphthalene), 1263 (=C—O—C) cm^-1^.

HRMS (*m/z*): [*M* + H]^+^ calcd for C_30_H_27_O_6_, 483.1808 found, 483.1836.

m.p. 537.5–538.8 K

### Refinement

All H atoms were put in calculated positions and treated as riding on their parent atoms, with C—H = 0.95(aromatic C—H), 0.98(methyl), 0.99(methylene) Å, and *U*_ĩso_(H) = 1.2 *U*_eq_(aromatic C, methyl C, methylene C). The positions of methyl hydrogens were rotationally optimized.

### Crystal data

**Table:**

C_30_H_26_O_6_·C_3_H_6_O	*D*_x_ = 1.280 Mg m^−^^3^
*M**_r_* = 540.59	Melting point = 537.5–538.8 K
Orthorhombic, *P**b**c**a*	Cu *K*α radiation, λ = 1.54187 Å
Hall symbol: -P 2ac 2ab	Cell parameters from 92773 reflections
*a* = 15.4948 (3) Å	θ = 3.4–68.3°
*b* = 16.1272 (3) Å	µ = 0.73 mm^−^^1^
*c* = 22.4430 (4) Å	*T* = 193 K
*V* = 5608.23 (18) Å^3^	Block, colorless
*Z* = 8	0.50 × 0.45 × 0.40 mm
*F*(000) = 2288	

### Data collection

**Table:**

Rigaku R-AXIS RAPID diffractometer	5132 independent reflections
Radiation source: rotating anode	4829 reflections with *I* > 2σ(*I*)
Graphite monochromator	*R*_int_ = 0.021
Detector resolution: 10.000 pixels mm^-1^	θ_max_ = 68.3°, θ_min_ = 3.9°
ω scans	*h* = −18→18
Absorption correction: numerical (*NUMABS*; Higashi, 1999)	*k* = −19→19
*T*_min_ = 0.712, *T*_max_ = 0.759	*l* = −27→27
99441 measured reflections	

### Refinement

**Table:**

Refinement on *F*^2^	Secondary atom site location: difference Fourier map
Least-squares matrix: full	Hydrogen site location: inferred from neighbouring sites
*R*[*F*^2^ > 2σ(*F*^2^)] = 0.037	H-atom parameters constrained
*wR*(*F*^2^) = 0.098	*w* = 1/[σ^2^(*F*_o_^2^) + (0.0495*P*)^2^ + 1.7635*P*] where *P* = (*F*_o_^2^ + 2*F*_c_^2^)/3
*S* = 1.05	(Δ/σ)_max_ = 0.001
5132 reflections	Δρ_max_ = 0.20 e Å^−^^3^
366 parameters	Δρ_min_ = −0.19 e Å^−^^3^
0 restraints	Extinction correction: *SHELXL97* (Sheldrick, 2008), Fc^*^=kFc[1+0.001xFc^2^λ^3^/6(2θ)]^-1/4^
Primary atom site location: structure-invariant direct methods	Extinction coefficient: 0.00160 (9)

### Special details

**Table:**

Geometry. All e.s.d.'s (except the e.s.d. in the dihedral angle between two l.s. planes) are estimated using the full covariance matrix. The cell e.s.d.'s are taken into account individually in the estimation of e.s.d.'s in distances, angles and torsion angles; correlations between e.s.d.'s in cell parameters are only used when they are defined by crystal symmetry. An approximate (isotropic) treatment of cell e.s.d.'s is used for estimating e.s.d.'s involving l.s. planes.
Refinement. Refinement of *F*^2^ against ALL reflections. The weighted *R*-factor *wR* and goodness of fit *S* are based on *F*^2^, conventional *R*-factors *R* are based on *F*, with *F* set to zero for negative *F*^2^. The threshold expression of *F*^2^ > σ(*F*^2^) is used only for calculating *R*-factors(gt) *etc*. and is not relevant to the choice of reflections for refinement. *R*-factors based on *F*^2^ are statistically about twice as large as those based on *F*, and *R*- factors based on ALL data will be even larger.

### Fractional atomic coordinates and isotropic or equivalent isotropic displacement parameters (Å^2^)

**Table:**

	*x*	*y*	*z*	*U*_iso_*/*U*_eq_	
O1	1.07047 (5)	0.27102 (5)	0.20944 (4)	0.0347 (2)	
O2	1.19956 (5)	0.17013 (6)	0.14801 (4)	0.0365 (2)	
O3	0.85352 (6)	0.25079 (6)	0.23241 (4)	0.0408 (2)	
O4	1.19863 (7)	−0.01523 (6)	0.13445 (5)	0.0546 (3)	
O5	0.89572 (6)	0.42949 (5)	−0.01715 (4)	0.0411 (2)	
O6	1.01931 (7)	0.19019 (6)	−0.10484 (4)	0.0521 (3)	
O1S	0.72978 (11)	0.11706 (9)	0.09895 (8)	0.0950 (5)	
C1	0.96632 (7)	0.16581 (7)	0.20049 (5)	0.0284 (2)	
C2	0.88697 (8)	0.17239 (7)	0.22823 (5)	0.0324 (3)	
C3	0.84625 (8)	0.10363 (8)	0.25505 (5)	0.0378 (3)	
H3	0.7913	0.1093	0.2734	0.045*	
C4	0.88732 (9)	0.02911 (8)	0.25413 (6)	0.0392 (3)	
H4	0.8608	−0.0171	0.2729	0.047*	
C5	0.96831 (8)	0.01832 (8)	0.22606 (5)	0.0351 (3)	
C6	1.00846 (10)	−0.06012 (8)	0.22537 (6)	0.0434 (3)	
H6	0.9814	−0.1052	0.2452	0.052*	
C7	1.08489 (10)	−0.07290 (8)	0.19709 (7)	0.0463 (3)	
H7	1.1115	−0.1260	0.1978	0.056*	
C8	1.12400 (9)	−0.00658 (8)	0.16673 (6)	0.0400 (3)	
C9	1.08822 (8)	0.07214 (7)	0.16682 (5)	0.0315 (3)	
C10	1.00922 (8)	0.08721 (7)	0.19760 (5)	0.0295 (3)	
C11	1.00809 (7)	0.24613 (7)	0.18132 (5)	0.0274 (2)	
C12	0.97305 (7)	0.29312 (7)	0.13029 (5)	0.0276 (2)	
C13	1.01308 (8)	0.36739 (7)	0.11391 (5)	0.0319 (3)	
H13	1.0598	0.3879	0.1371	0.038*	
C14	0.98588 (8)	0.41120 (7)	0.06475 (6)	0.0349 (3)	
H14	1.0139	0.4614	0.0539	0.042*	
C15	0.91706 (8)	0.38176 (7)	0.03089 (5)	0.0328 (3)	
C16	0.87467 (8)	0.30933 (8)	0.04747 (6)	0.0341 (3)	
H16	0.8264	0.2902	0.0253	0.041*	
C17	0.90344 (8)	0.26532 (7)	0.09658 (5)	0.0312 (3)	
H17	0.8752	0.2153	0.1074	0.037*	
C18	1.13496 (7)	0.13598 (7)	0.12938 (5)	0.0293 (2)	
C19	1.10244 (7)	0.15208 (7)	0.06846 (5)	0.0299 (3)	
C20	1.13965 (8)	0.21529 (7)	0.03479 (6)	0.0334 (3)	
H20	1.1838	0.2483	0.0520	0.040*	
C21	1.11380 (8)	0.23109 (8)	−0.02314 (6)	0.0385 (3)	
H21	1.1399	0.2744	−0.0455	0.046*	
C22	1.04891 (9)	0.18246 (8)	−0.04813 (6)	0.0394 (3)	
C23	1.01050 (9)	0.11965 (8)	−0.01486 (6)	0.0414 (3)	
H23	0.9659	0.0870	−0.0320	0.050*	
C24	1.03688 (8)	0.10465 (7)	0.04282 (6)	0.0361 (3)	
H24	1.0103	0.0617	0.0652	0.043*	
C25	0.76197 (9)	0.25914 (10)	0.22669 (7)	0.0496 (4)	
H25A	0.7472	0.3177	0.2212	0.060*	
H25B	0.7339	0.2382	0.2628	0.060*	
H25C	0.7421	0.2272	0.1922	0.060*	
C26	1.22703 (12)	−0.09660 (10)	0.12000 (10)	0.0656 (5)	
H26A	1.1788	−0.1284	0.1034	0.079*	
H26B	1.2482	−0.1241	0.1561	0.079*	
H26C	1.2737	−0.0934	0.0906	0.079*	
C27	0.84853 (8)	0.39230 (9)	−0.06580 (6)	0.0410 (3)	
H27A	0.8372	0.4352	−0.0964	0.049*	
H27B	0.7921	0.3725	−0.0508	0.049*	
C28	0.89555 (9)	0.32062 (9)	−0.09454 (6)	0.0409 (3)	
H28A	0.8645	0.3045	−0.1313	0.049*	
H28B	0.8942	0.2726	−0.0671	0.049*	
C29	0.98906 (9)	0.33975 (9)	−0.11025 (6)	0.0435 (3)	
H29A	0.9906	0.3864	−0.1389	0.052*	
H29B	1.0199	0.3574	−0.0738	0.052*	
C30	1.03545 (11)	0.26622 (10)	−0.13715 (6)	0.0531 (4)	
H30A	1.0165	0.2592	−0.1790	0.064*	
H30B	1.0983	0.2774	−0.1374	0.064*	
C1S	0.73688 (11)	0.04533 (11)	0.11251 (9)	0.0641 (4)	
C2S	0.68285 (14)	0.00854 (15)	0.16044 (10)	0.0800 (6)	
H2S1	0.7202	−0.0168	0.1906	0.096*	
H2S2	0.6450	−0.0339	0.1434	0.096*	
H2S3	0.6477	0.0520	0.1789	0.096*	
C3S	0.80147 (16)	−0.00945 (16)	0.08399 (14)	0.1037 (8)	
H3S1	0.8474	−0.0222	0.1125	0.124*	
H3S2	0.8262	0.0186	0.0492	0.124*	
H3S3	0.7735	−0.0610	0.0713	0.124*	

### Atomic displacement parameters (Å^2^)

**Table:**

	*U*^11^	*U*^22^	*U*^33^	*U*^12^	*U*^13^	*U*^23^
O1	0.0305 (4)	0.0346 (4)	0.0390 (5)	−0.0018 (3)	−0.0044 (4)	−0.0020 (3)
O2	0.0293 (4)	0.0451 (5)	0.0350 (5)	−0.0036 (4)	−0.0012 (4)	−0.0012 (4)
O3	0.0337 (5)	0.0391 (5)	0.0494 (5)	−0.0001 (4)	0.0108 (4)	−0.0077 (4)
O4	0.0438 (6)	0.0353 (5)	0.0847 (8)	0.0101 (4)	0.0133 (5)	−0.0011 (5)
O5	0.0472 (5)	0.0364 (5)	0.0398 (5)	−0.0068 (4)	−0.0094 (4)	0.0096 (4)
O6	0.0694 (7)	0.0524 (6)	0.0345 (5)	0.0088 (5)	−0.0144 (5)	−0.0026 (4)
O1S	0.0980 (11)	0.0695 (9)	0.1174 (13)	0.0063 (8)	−0.0003 (10)	0.0117 (9)
C1	0.0308 (6)	0.0314 (6)	0.0229 (5)	−0.0028 (5)	−0.0010 (4)	0.0000 (4)
C2	0.0335 (6)	0.0363 (6)	0.0273 (6)	−0.0032 (5)	0.0012 (5)	−0.0037 (5)
C3	0.0359 (6)	0.0472 (7)	0.0304 (6)	−0.0097 (5)	0.0056 (5)	−0.0015 (5)
C4	0.0458 (7)	0.0416 (7)	0.0302 (6)	−0.0135 (6)	0.0016 (5)	0.0064 (5)
C5	0.0420 (7)	0.0343 (6)	0.0291 (6)	−0.0062 (5)	−0.0040 (5)	0.0051 (5)
C6	0.0535 (8)	0.0325 (6)	0.0441 (7)	−0.0062 (6)	−0.0064 (6)	0.0118 (6)
C7	0.0508 (8)	0.0294 (6)	0.0585 (9)	0.0048 (6)	−0.0075 (7)	0.0076 (6)
C8	0.0361 (7)	0.0342 (6)	0.0497 (8)	0.0037 (5)	−0.0038 (6)	0.0016 (6)
C9	0.0319 (6)	0.0304 (6)	0.0322 (6)	0.0010 (5)	−0.0044 (5)	0.0022 (5)
C10	0.0328 (6)	0.0309 (6)	0.0247 (5)	−0.0023 (5)	−0.0048 (4)	0.0025 (4)
C11	0.0263 (5)	0.0274 (5)	0.0284 (6)	0.0015 (4)	0.0039 (4)	−0.0052 (4)
C12	0.0285 (6)	0.0261 (5)	0.0282 (6)	0.0000 (4)	0.0038 (4)	−0.0022 (4)
C13	0.0323 (6)	0.0307 (6)	0.0326 (6)	−0.0056 (5)	−0.0011 (5)	−0.0026 (5)
C14	0.0393 (7)	0.0287 (6)	0.0367 (6)	−0.0078 (5)	0.0006 (5)	0.0019 (5)
C15	0.0347 (6)	0.0316 (6)	0.0319 (6)	0.0003 (5)	0.0008 (5)	0.0027 (5)
C16	0.0314 (6)	0.0364 (6)	0.0345 (6)	−0.0063 (5)	−0.0037 (5)	0.0005 (5)
C17	0.0328 (6)	0.0285 (6)	0.0325 (6)	−0.0056 (5)	0.0030 (5)	0.0006 (5)
C18	0.0260 (5)	0.0284 (6)	0.0334 (6)	0.0047 (4)	0.0024 (5)	−0.0027 (5)
C19	0.0274 (6)	0.0290 (6)	0.0334 (6)	0.0048 (4)	0.0003 (5)	−0.0021 (5)
C20	0.0288 (6)	0.0340 (6)	0.0373 (6)	0.0015 (5)	−0.0015 (5)	0.0003 (5)
C21	0.0378 (7)	0.0398 (7)	0.0377 (7)	0.0046 (5)	0.0009 (5)	0.0068 (5)
C22	0.0444 (7)	0.0412 (7)	0.0327 (6)	0.0116 (6)	−0.0060 (5)	−0.0047 (5)
C23	0.0449 (7)	0.0357 (7)	0.0436 (7)	0.0001 (6)	−0.0114 (6)	−0.0072 (5)
C24	0.0368 (6)	0.0306 (6)	0.0410 (7)	0.0003 (5)	−0.0035 (5)	−0.0012 (5)
C25	0.0373 (7)	0.0528 (8)	0.0588 (9)	0.0057 (6)	0.0047 (6)	0.0065 (7)
C26	0.0530 (9)	0.0409 (8)	0.1029 (14)	0.0106 (7)	0.0068 (9)	−0.0143 (9)
C27	0.0364 (7)	0.0479 (7)	0.0387 (7)	−0.0037 (6)	−0.0100 (5)	0.0098 (6)
C28	0.0409 (7)	0.0469 (7)	0.0350 (7)	−0.0063 (6)	−0.0092 (5)	0.0073 (6)
C29	0.0406 (7)	0.0512 (8)	0.0388 (7)	−0.0013 (6)	−0.0050 (6)	0.0134 (6)
C30	0.0586 (9)	0.0698 (10)	0.0309 (7)	0.0094 (8)	−0.0038 (6)	0.0079 (7)
C1S	0.0513 (9)	0.0580 (10)	0.0830 (12)	−0.0041 (8)	−0.0127 (9)	−0.0068 (9)
C2S	0.0652 (12)	0.0906 (14)	0.0844 (14)	−0.0182 (10)	−0.0137 (10)	0.0009 (11)
C3S	0.0761 (15)	0.0973 (17)	0.138 (2)	0.0054 (13)	0.0116 (15)	−0.0289 (16)

### Geometric parameters (Å, º)

**Table:**

O1—C11	1.2222 (14)	C17—H17	0.9500
O2—C18	1.2166 (14)	C18—C19	1.4800 (16)
O3—C2	1.3697 (15)	C19—C20	1.3939 (17)
O3—C25	1.4306 (16)	C19—C24	1.3959 (17)
O4—C8	1.3717 (17)	C20—C21	1.3841 (18)
O4—C26	1.4215 (17)	C20—H20	0.9500
O5—C15	1.3654 (15)	C21—C22	1.3930 (19)
O5—C27	1.4445 (16)	C21—H21	0.9500
O6—C22	1.3586 (16)	C22—C23	1.392 (2)
O6—C30	1.4463 (19)	C23—C24	1.3787 (18)
O1S—C1S	1.201 (2)	C23—H23	0.9500
C1—C2	1.3822 (16)	C24—H24	0.9500
C1—C10	1.4328 (16)	C25—H25A	0.9800
C1—C11	1.5107 (15)	C25—H25B	0.9800
C2—C3	1.4109 (17)	C25—H25C	0.9800
C3—C4	1.3600 (19)	C26—H26A	0.9800
C3—H3	0.9500	C26—H26B	0.9800
C4—C5	1.4149 (19)	C26—H26C	0.9800
C4—H4	0.9500	C27—C28	1.511 (2)
C5—C6	1.4098 (19)	C27—H27A	0.9900
C5—C10	1.4297 (16)	C27—H27B	0.9900
C6—C7	1.359 (2)	C28—C29	1.5227 (19)
C6—H6	0.9500	C28—H28A	0.9900
C7—C8	1.4057 (19)	C28—H28B	0.9900
C7—H7	0.9500	C29—C30	1.512 (2)
C8—C9	1.3852 (17)	C29—H29A	0.9900
C9—C10	1.4266 (17)	C29—H29B	0.9900
C9—C18	1.5134 (16)	C30—H30A	0.9900
C11—C12	1.4766 (16)	C30—H30B	0.9900
C12—C17	1.3917 (16)	C1S—C3S	1.481 (3)
C12—C13	1.3980 (16)	C1S—C2S	1.487 (3)
C13—C14	1.3764 (17)	C2S—H2S1	0.9800
C13—H13	0.9500	C2S—H2S2	0.9800
C14—C15	1.3927 (17)	C2S—H2S3	0.9800
C14—H14	0.9500	C3S—H3S1	0.9800
C15—C16	1.3908 (17)	C3S—H3S2	0.9800
C16—C17	1.3846 (17)	C3S—H3S3	0.9800
C16—H16	0.9500		
			
C2—O3—C25	117.13 (10)	C19—C20—H20	119.2
C8—O4—C26	118.39 (12)	C20—C21—C22	118.91 (12)
C15—O5—C27	119.04 (10)	C20—C21—H21	120.5
C22—O6—C30	119.28 (11)	C22—C21—H21	120.5
C2—C1—C10	120.07 (10)	O6—C22—C23	115.15 (12)
C2—C1—C11	116.33 (10)	O6—C22—C21	124.69 (13)
C10—C1—C11	123.16 (10)	C23—C22—C21	120.15 (12)
O3—C2—C1	116.00 (10)	C24—C23—C22	120.31 (12)
O3—C2—C3	121.81 (11)	C24—C23—H23	119.8
C1—C2—C3	121.98 (11)	C22—C23—H23	119.8
C4—C3—C2	118.61 (12)	C23—C24—C19	120.44 (12)
C4—C3—H3	120.7	C23—C24—H24	119.8
C2—C3—H3	120.7	C19—C24—H24	119.8
C3—C4—C5	122.04 (11)	O3—C25—H25A	109.5
C3—C4—H4	119.0	O3—C25—H25B	109.5
C5—C4—H4	119.0	H25A—C25—H25B	109.5
C6—C5—C4	120.45 (12)	O3—C25—H25C	109.5
C6—C5—C10	119.78 (12)	H25A—C25—H25C	109.5
C4—C5—C10	119.77 (11)	H25B—C25—H25C	109.5
C7—C6—C5	121.72 (12)	O4—C26—H26A	109.5
C7—C6—H6	119.1	O4—C26—H26B	109.5
C5—C6—H6	119.1	H26A—C26—H26B	109.5
C6—C7—C8	119.11 (12)	O4—C26—H26C	109.5
C6—C7—H7	120.4	H26A—C26—H26C	109.5
C8—C7—H7	120.4	H26B—C26—H26C	109.5
O4—C8—C9	115.56 (11)	O5—C27—C28	113.35 (10)
O4—C8—C7	122.82 (12)	O5—C27—H27A	108.9
C9—C8—C7	121.61 (13)	C28—C27—H27A	108.9
C8—C9—C10	120.01 (11)	O5—C27—H27B	108.9
C8—C9—C18	115.55 (11)	C28—C27—H27B	108.9
C10—C9—C18	124.31 (10)	H27A—C27—H27B	107.7
C9—C10—C5	117.68 (11)	C27—C28—C29	113.75 (11)
C9—C10—C1	124.80 (10)	C27—C28—H28A	108.8
C5—C10—C1	117.49 (11)	C29—C28—H28A	108.8
O1—C11—C12	121.52 (10)	C27—C28—H28B	108.8
O1—C11—C1	118.25 (10)	C29—C28—H28B	108.8
C12—C11—C1	120.23 (10)	H28A—C28—H28B	107.7
C17—C12—C13	118.49 (11)	C30—C29—C28	112.69 (13)
C17—C12—C11	122.76 (10)	C30—C29—H29A	109.1
C13—C12—C11	118.72 (10)	C28—C29—H29A	109.1
C14—C13—C12	120.98 (11)	C30—C29—H29B	109.1
C14—C13—H13	119.5	C28—C29—H29B	109.1
C12—C13—H13	119.5	H29A—C29—H29B	107.8
C13—C14—C15	119.79 (11)	O6—C30—C29	112.49 (12)
C13—C14—H14	120.1	O6—C30—H30A	109.1
C15—C14—H14	120.1	C29—C30—H30A	109.1
O5—C15—C16	124.78 (11)	O6—C30—H30B	109.1
O5—C15—C14	115.09 (10)	C29—C30—H30B	109.1
C16—C15—C14	120.13 (11)	H30A—C30—H30B	107.8
C17—C16—C15	119.44 (11)	O1S—C1S—C3S	121.8 (2)
C17—C16—H16	120.3	O1S—C1S—C2S	121.1 (2)
C15—C16—H16	120.3	C3S—C1S—C2S	117.1 (2)
C16—C17—C12	121.13 (11)	C1S—C2S—H2S1	109.5
C16—C17—H17	119.4	C1S—C2S—H2S2	109.5
C12—C17—H17	119.4	H2S1—C2S—H2S2	109.5
O2—C18—C19	121.20 (11)	C1S—C2S—H2S3	109.5
O2—C18—C9	120.73 (10)	H2S1—C2S—H2S3	109.5
C19—C18—C9	117.99 (10)	H2S2—C2S—H2S3	109.5
C20—C19—C24	118.57 (11)	C1S—C3S—H3S1	109.5
C20—C19—C18	119.23 (11)	H3S1—C3S—H3S2	109.5
C24—C19—C18	122.18 (11)	H3S1—C3S—H3S3	109.5
C21—C20—C19	121.61 (12)	H3S2—C3S—H3S3	109.5
C21—C20—H20	119.2		
			
C25—O3—C2—C1	144.21 (12)	O1—C11—C12—C13	0.48 (16)
C25—O3—C2—C3	−40.90 (17)	C1—C11—C12—C13	179.74 (10)
C10—C1—C2—O3	175.75 (10)	C17—C12—C13—C14	−1.57 (17)
C11—C1—C2—O3	3.16 (15)	C11—C12—C13—C14	176.45 (11)
C10—C1—C2—C3	0.87 (17)	C12—C13—C14—C15	0.49 (18)
C11—C1—C2—C3	−171.72 (11)	C27—O5—C15—C16	−22.73 (18)
O3—C2—C3—C4	−173.61 (11)	C27—O5—C15—C14	158.29 (11)
C1—C2—C3—C4	0.97 (18)	C13—C14—C15—O5	−179.49 (11)
C2—C3—C4—C5	−1.43 (19)	C13—C14—C15—C16	1.47 (19)
C3—C4—C5—C6	−179.21 (12)	O5—C15—C16—C17	178.76 (12)
C3—C4—C5—C10	0.05 (19)	C14—C15—C16—C17	−2.30 (19)
C4—C5—C6—C7	177.84 (13)	C15—C16—C17—C12	1.20 (18)
C10—C5—C6—C7	−1.4 (2)	C13—C12—C17—C16	0.71 (17)
C5—C6—C7—C8	−1.3 (2)	C11—C12—C17—C16	−177.22 (11)
C26—O4—C8—C9	−164.56 (14)	C8—C9—C18—O2	−80.03 (15)
C26—O4—C8—C7	14.4 (2)	C10—C9—C18—O2	104.23 (14)
C6—C7—C8—O4	−176.42 (13)	C8—C9—C18—C19	96.73 (13)
C6—C7—C8—C9	2.5 (2)	C10—C9—C18—C19	−79.01 (14)
O4—C8—C9—C10	178.17 (11)	O2—C18—C19—C20	−8.52 (16)
C7—C8—C9—C10	−0.8 (2)	C9—C18—C19—C20	174.74 (10)
O4—C8—C9—C18	2.23 (17)	O2—C18—C19—C24	169.87 (11)
C7—C8—C9—C18	−176.74 (12)	C9—C18—C19—C24	−6.87 (16)
C8—C9—C10—C5	−1.91 (17)	C24—C19—C20—C21	−0.75 (18)
C18—C9—C10—C5	173.65 (11)	C18—C19—C20—C21	177.70 (11)
C8—C9—C10—C1	−179.77 (11)	C19—C20—C21—C22	0.07 (18)
C18—C9—C10—C1	−4.20 (18)	C30—O6—C22—C23	162.23 (12)
C6—C5—C10—C9	3.00 (17)	C30—O6—C22—C21	−18.9 (2)
C4—C5—C10—C9	−176.27 (11)	C20—C21—C22—O6	−178.25 (12)
C6—C5—C10—C1	−178.99 (11)	C20—C21—C22—C23	0.59 (19)
C4—C5—C10—C1	1.75 (16)	O6—C22—C23—C24	178.38 (12)
C2—C1—C10—C9	175.67 (11)	C21—C22—C23—C24	−0.6 (2)
C11—C1—C10—C9	−12.26 (17)	C22—C23—C24—C19	−0.13 (19)
C2—C1—C10—C5	−2.18 (16)	C20—C19—C24—C23	0.77 (18)
C11—C1—C10—C5	169.88 (10)	C18—C19—C24—C23	−177.63 (11)
C2—C1—C11—O1	107.36 (12)	C15—O5—C27—C28	−59.83 (15)
C10—C1—C11—O1	−64.98 (14)	O5—C27—C28—C29	−49.10 (15)
C2—C1—C11—C12	−71.92 (13)	C27—C28—C29—C30	178.19 (11)
C10—C1—C11—C12	115.74 (12)	C22—O6—C30—C29	−65.93 (17)
O1—C11—C12—C17	178.41 (11)	C28—C29—C30—O6	−45.27 (16)
C1—C11—C12—C17	−2.33 (16)		

### Hydrogen-bond geometry (Å, º)

Cg is the centroid of the C12–C17 ring.

**Table:**

*D*—H···*A*	*D*—H	H···*A*	*D*···*A*	*D*—H···*A*
C3—H3···O2^i^	0.95	2.47	3.3241 (15)	150
C6—H6···O1^ii^	0.95	2.38	3.3245 (16)	172
C7—H7···O3^ii^	0.95	2.59	3.3910 (17)	143
C14—H14···O5^iii^	0.95	2.40	3.3328 (15)	169
C21—H21···O1*S*^iv^	0.95	2.54	3.482 (2)	172
C2*S*—H2*S*2···*Cg*^v^	0.98	2.86	3.830 (2)	171

Symmetry codes: (i) *x*−1/2, *y*, −*z*+1/2; (ii) −*x*+2, *y*−1/2, −*z*+1/2; (iii) −*x*+2, −*y*+1, −*z*; (iv) *x*+1/2, −*y*+1/2, −*z*; (v) −*x*+3/2, *y*−1/2, *z*.
